# Anti-*Trichomonas vaginalis* Activity of Triterpenes from *Tagetes nelsonii* Greenm

**DOI:** 10.3390/ph18101587

**Published:** 2025-10-21

**Authors:** Mario Alberto Hernández-Torres, Sara García-Davis, José J. Fernández, Ana R. Diaz-Marrero, Magda Elizabeth Hernández-García, Irma Edith Carranza-Torres, Ezequiel Viveros-Valdez

**Affiliations:** 1 Departamento de Química, Facultad de Ciencias Biológicas, Universidad Autónoma de Nuevo León, Avda. Pedro de Alba S/N, San Nicolás de los Garza 66450, Nuevo León, Mexico; mhernandezt@uanl.edu.mx (M.A.H.-T.); irma.carranzatr@uanl.edu.mx (I.E.C.-T.); 2 Instituto Universitario de Bio-Orgánica Antonio González (IUBO AG), Universidad de La Laguna (ULL), Avda. Astrofísico F. Sánchez, 2, 38206 La Laguna, Spain; sgdavis@ull.edu.es (S.G.-D.); jjfercas@ull.edu.es (J.J.F.); 3 Biotecnología Marina, Unidad Asociada al IPNA-CSIC por el IUBO-ULL, 38206 La Laguna, Spain; adiazmar@ipna.csic.es; 4 Instituto de Productos Naturales y Agrobiología (IPNA), Consejo Superior de Investigaciones Científicas (CSIC), Avenida Astrofísico Francisco Sánchez 3, 38206 La Laguna, Spain; 5 Instituto Mexicano del Seguro Social, Centro de Investigación Biomédicas del Noreste, 2 de Abril # 501, Monterrey 64720, Nuevo León, Mexico; magda.hernandezg@imss.gob.mx

**Keywords:** *Tagetes nelsonii*, triterpenes, betulin, stigmasterol, anti-trichomonal activity, molecular docking

## Abstract

**Background: ***Trichomonas vaginalis* is the causative agent of human trichomoniasis, the most common non-viral sexually transmitted infection. This disease is associated with an increased susceptibility to HIV and HPV infections. Currently, resistance to metronidazole (MTZ), the main drug used for treatment, has been reported in up to 9.6% of cases; additionally, the compound is also associated with adverse side effects. Therefore, it is urgent to identify new treatment options. **Objective**: In this study, we investigated for the first time the in vitro and in silico activity against *T. vaginalis* of betulin and stigmasterol isolated from *Tagetes nelsonii* Greenm, as well as their hemolytic activity. **Methods:** Plant specimen was collected in Chiapas, Mexico. Hexane and methanol extracts were prepared through sonication-assisted maceration. The antiprotozoal and hemolytic activities were evaluated in vitro against *Trichomonas vaginalis* trophozoites and human erythrocytes. The most active extract was fractionated using chromatographic techniques in a bioassay-guided study. The active metabolites were identified by ^1^H and ^13^C-NMR spectroscopy, and their biological activity was further assessed in silico against lactate dehydrogenase (LDH), pyruvate ferredoxin oxidoreductase (PFOR) methionine gamma-lyase (MGL) and purine nucleoside phosphorylase (PNP) *T. vaginalis* enzymes. **Results:** Both triterpenes showed anti-trichomonal activity and no hemolytic activity at 100 µg/mL. Molecular docking studies predicted promising interactions of triterpenes with *T. vaginalis* drug target proteins, TvpFOR and TvLDH. **Conclusions:** Our results revealed that betulin and stigmasterol are potential molecules for the development of new trichomonacidal therapies against *T. vaginalis*.

## 1. Introduction

*Trichomonas vaginalis* is an anaerobic, flagellated protozoan parasite that infects the human urogenital tract, causing trichomoniasis, the most common non-viral sexually transmitted infection globally, with approximately 156 million cases per year [1]. Trichomoniasis represents a serious global reproductive health concern. Approximately 30% of infected individuals, predominantly women, develop symptoms such as genital discomfort, itching, burning, odor and discharge [2,3,4]. Chronic or untreated infections have been associated with an increased risk of human papillomavirus (HPV) and human immunodeficiency virus (HIV) infections, pelvic inflammatory disease, cervical neoplasia, adverse pregnancy outcomes, and infertility [5,6,7].

Current treatment for *Trichomonas vaginalis* infection relies almost exclusively on 5-nitroimidazole (5-NMZ) derivatives, mainly metronidazole (MTZ) and tinidazole (TNZ). Nevertheless, the emergence of resistant strains, reported in up to 9.6% of clinical isolates [8], together with documented genotoxic and neurotoxic side effects of MTZ, including encephalopathy [9,10,11,12], highlight the urgent need for novel and safer therapeutic alternatives to manage trichomoniasis.

Natural products represent a prolific source of structurally diverse metabolites capable of modulating multiple cellular processes in protozoa, including redox homeostasis, membrane integrity, and mitochondrial/hydrogenosomal metabolism. In this context, medicinal plants offer key advantages such as well-established safety profiles, potent pharmacological effects, cost-effectiveness, and extensive traditional use in humans and animals. Consequently, the World Health Organization (WHO) has endorsed the integration of herbal medicines within traditional healthcare systems [13].

In recent years, multiple studies have revealed the potential of natural products as promising alternative treatments for trichomoniasis, especially those derived from ethnomedicinal plants [14]. Furthermore, several anti-*T. vaginalis* compounds have also been isolated from microorganisms and other natural sources [15].

Bioactive metabolites with reported trichomonacidal activity include ajoene and allicin from *Allium sativum*, berberine from *Berberis aristata*, resveratrol and catechins from grapes and *Camellia sinensis*, as well as cinnamaldehyde, carvacrol, and thymol from *Cinnamomum zeylanicum* [16,17,18,19,20,21,22]. In addition, fungal metabolites such as phomasetin and pyrrolocin A—particularly tetramate derivatives—have also shown promising in vitro antiparasitic effects [23]. Vaginal *Lactobacillus* species also inhibit *T. vaginalis* through the production of lactic acid, hydrogen peroxide, and bacteriocin-like peptides [24].

Despite extensive research into natural antiparasitic agents, their translation into clinically approved drugs remains limited. Since 1981, only about twenty natural products or derivatives have been approved by the U.S. Food and Drug Administration (FDA) [25]. Therefore, the continued exploration of natural sources for antiparasitic drug discovery remains a relevant and promising strategy.

The genus *Tagetes* (Asteraceae) comprises a diverse group of herbaceous plants commonly known as “cempasúchil,” “clavel de muerto,” or “marigold.” Native to the Americas, Mexico is recognized as the center of origin and diversity for these aromatic and visually striking species. Several *Tagetes* species are cultivated worldwide and used in traditional medicine for their wide range of therapeutic properties including applications in skin care, anti-inflammatory, analgesic, antispasmodic, antipyretic, dental healing, antidiarrheal, anti-abortifacient, and vaginal antiseptic treatments [26,27,28,29,30]. In particular, our research group has previously reported anti-*Trichomonas tenax* activity in *Tagetes nelsonii*, a protozoan associated with periodontal disease [31].

Phytochemical analyses of *Tagetes* species have revealed a rich diversity of metabolites with pharmacological potential, notably monoterpenes, phenylpropanoids, carotenoids, flavonoids, and thiophenes [32]. The biological activities of these constituents have been extensively documented, including antimicrobial effects against bacteria, fungi, and protozoa [33,34,35], as well as insecticidal [36,37,38], nematicidal [39,40], antioxidant, antineoplastic [41], and cytotoxic properties [42,43].

Ethnomedicinal knowledge has proven valuable tool for guiding the systematic screening of plants with antiprotozoal potential. Building upon previous evidence of biological activity in *Tagetes* species, this study explored the in vitro and in silico anti-*Trichomonas vaginalis* activity of betulin and stigmasterol isolated from *Tagetes nelsonii*, a medicinal plant traditionally used in southeastern Mexico.

## 2. Results

### 2.1. Bioguided Isolation of Compounds from Tagetes nelsonii

**Figure 1:**
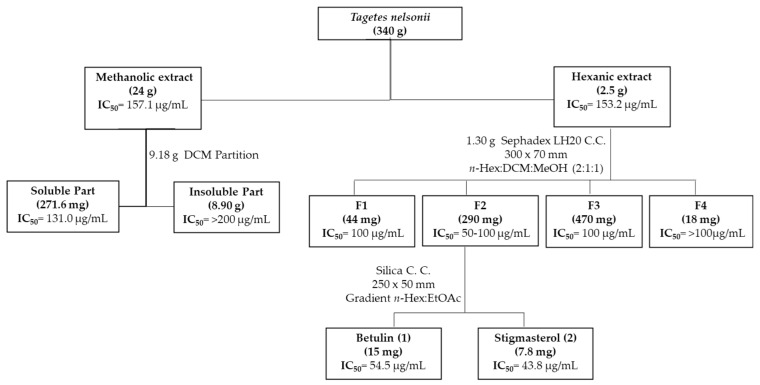
General scheme of the bioguided isolation of compounds with anti-*T. vaginalis* activity from *Tagetes nelsonii*.

The methanolic extract of *Tagetes nelsonii* was partitioned using dichloromethane (CH_2_Cl_2_), yielding a non-polar fraction that exhibited anti-*T. vaginalis* activity, suggesting that the active compounds were of low polarity. Subsequent fractionation of the hexanic extract by Sephadex column chromatography yielded fraction 2 with the best anti-trichomonal activity. Further purification of this active fraction by silica gel column chromatography led to the isolation of two known compounds, betulin (**1**) and stigmasterol (**2**), obtained as white powders, Figure 1.

### 2.2. Structural Elucidation of Isolated Compounds from Tagetes nelsonii

1D and 2D NMR spectra were recorded for the isolated compounds using deuterated chloroform (Table 1). The ^1^H-NMR spectra of compound **1** showed the presence of six methyl singlets at δ_H_ 1.03 (CH_3_, C-23), 0.94 (CH_3_, C-24), 0.83 (CH_3_, C-25), 1.01 (CH_3_, C-26), 0.97 (CH_3_, C-27) and 1.65 (CH_3_, C-30); the proton signal at δ_H_ 2.37 (CH, C-19, td, *J =* 5.9, 11.1, 11.1); one oxymethine proton at C-3 as a broad double doublet (δ_H_ 3.18, *J =* 4.7, 11.6 Hz) and proton doublet at δ_H_ 4.69 (CH_2_, C-29, *J =* 2.5 Hz) and double doublet 4.57 (CH_2_, C29, *J =* 1.5, 2.7 Hz) suggested a lupine-type triterpene. The ^13^C-NMR indicated the presence of an isopropenyl group with an olefinic quaternary carbon at δ_C_ 150.9 (C-20) and methylene carbon at δ_C_ 109.2 (C-29); and a characteristic signal at δ_C_ 78.9 corresponded to a hydroxyl-bearing carbon (C-3).

Regarding compound **2**, the ^1^H-NMR spectra exhibited six methyl groups at δ_H_ 0.69 (CH_3_, C-18), 1.01 (CH_3_, C-19), 1.02 (CH_3_, C-21), 0.85 (CH_3_, C-26), 0.84 (CH_3_, C-27) and 0.80 (CH_3_, C-29), a double doublets signal for a methine proton (CH, C-6) was observed at δ_H_ 5.36 (*J =* 2.3, 5.1 Hz); two signals corresponding to olefinic protons (CH, C-22, C-23) of a double bond were observed at δ_H_ 5.15 (dd, *J =* 8.7, 15.2 Hz) and δ_H_ 5.02 (dd, *J =* 8.7, 15.2 Hz); and a multiplet signal at δ_H_ 3.52 was assigned to an oxymethine proton (CH, C-3). The ^13^C-NMR showed a characteristic signal at δ_C_ 71.7 corresponded to a carbon attached to hydroxyl group (C-3); two signals at δ_C_ 129.2 and 138.2 were assigned to the olefinic carbons C-23 and C-22, respectively. Signals attributable to sp^2^ carbons of the endocyclic double bond were observed in the downfield region at δ_C_ 121.6 (C-6) and 140.7 (C-5).

**Figure 2:**
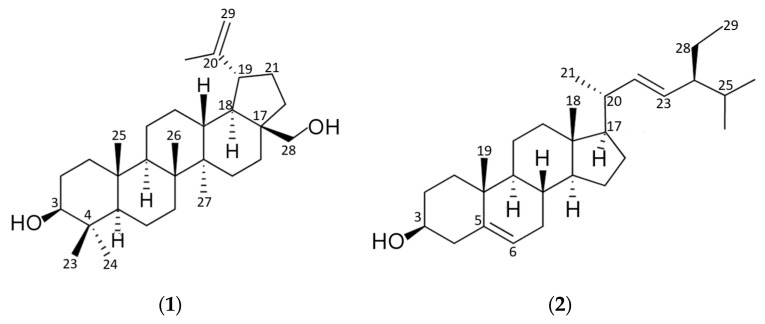
Molecular structure of triterpenoids isolated from *Tagetes nelsonii*: (**1**) betulin; (**2**) stigmasterol.

^1^H- and ^13^C-chemicals shifts of compounds **1** and **2** isolated from *Tagetes nelsonii* (Table 1) were in accordance with those reported for betulin (C_30_H_50_O_2_) [44,45] and stigmasterol (C_29_H_48_O) [46,47], respectively (Figure 2).

### 2.3. Anti-Trichomonas Vaginalis and Hemolytic Activity

According to previously established criteria for evaluating antiprotozoal activity, extracts and compounds with IC_50_ < 5 µg/mL are considered highly active, those with IC_50_ = 5–15 μg/mL exhibit promising activity, IC_50_ = 15–50 μg/mL indicate moderate activity, and IC_50_ > 50 μg/mL correspond to low or weak activity [48,49].

As shown in Table 2, methanolic and hexanic extracts from *Tagetes nelsonii* exhibited low anti-*Trichomonas vaginalis* activity, with no significate differences (Z = −0.535, *p* > 0.05). In contrast, the isolated compounds from hexanic extract showed moderate antiprotozoal activity (equal significance, Z = −1.604, *p* > 0.05), with stigmasterol exhibiting the strongest trichomonacidal activity (IC_50_ = 43.8 ± 4.3 μg/mL).

Regarding hemolytic activity, neither the extracts nor the isolated compounds induced erythrocyte lysis at the evaluated concentration.

### 2.4. In Silico Anti-Trichomonas Vaginalis Activity

A molecular docking study was carried out for betulin and stigmasterol to explore their potential mechanism of action based on predicted interactions with lactate dehydrogenase (LDH), pyruvate ferredoxin oxidoreductase (PFOR) methionine gamma-lyase (MGL) and purine nucleoside phosphorylase (PNP), all considered potential drug targets.

TvLDH and TvPFOR catalyze the oxidation of lactate and pyruvate, respectively, contributing to energy conservation through the generation of NADH molecules. Both enzymes play key roles in the carbohydrate metabolism of *T. vaginalis*. Notably, TvLDH exhibits low sequence similarity to human LDH, making it an attractive selective target [10,50]. The TvMGL enzyme catalyzes the catabolism of sulfur-containing amino acids and is essential for regulating their intracellular levels. Methionine γ-lyase (MGL) has been characterized in several bacterial species and in the parasitic protozoan *Entamoeba histolytica*, but it is absent in mammals [51]. In contrast, TvPNP catalyzes the interconversion between purine bases and purine nucleosides, functioning within the purine salvage pathway that is crucial for the survival of obligate parasitic protozoa [52].

Computational docking analysis revealed that both triterpenes interact favorably with TvPFOR, TvLDH, and TvPNP, displaying binding energies lower than −6.58 kcal/mol (Table 3) [53].

**Figure 3:**
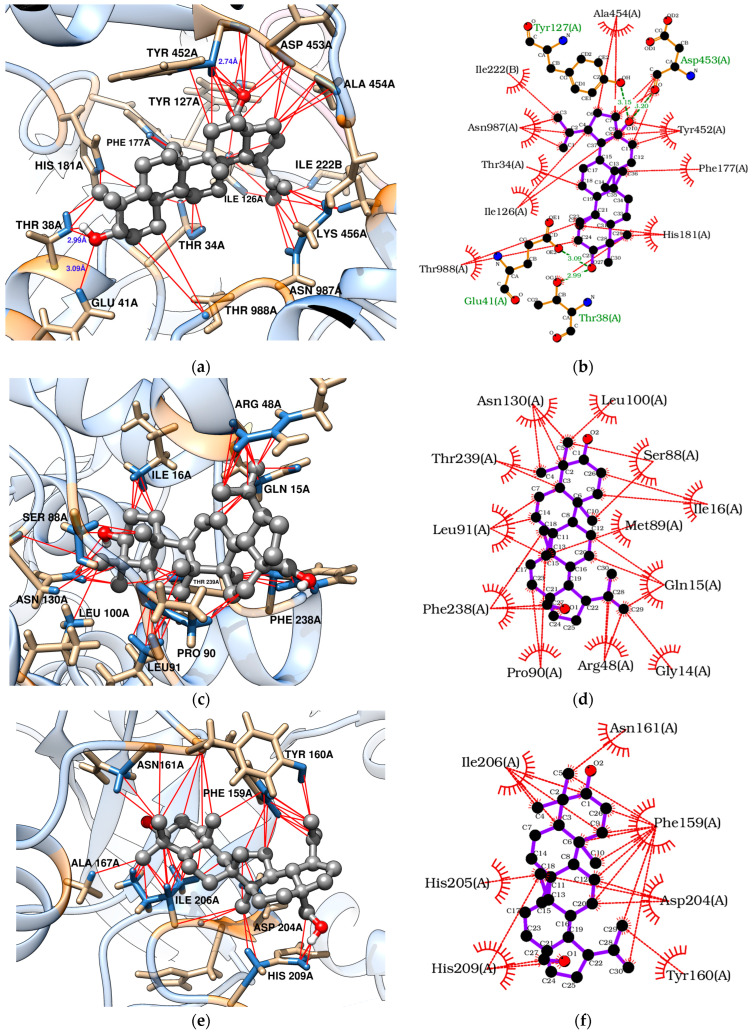
Molecular docking of betulin with *T.* vaginalis proteins: (**a**,**b**) TvpFOR; (**c**,**d**) TvLDH; (**e**,**f**) TvPNP.

**Figure 4:**
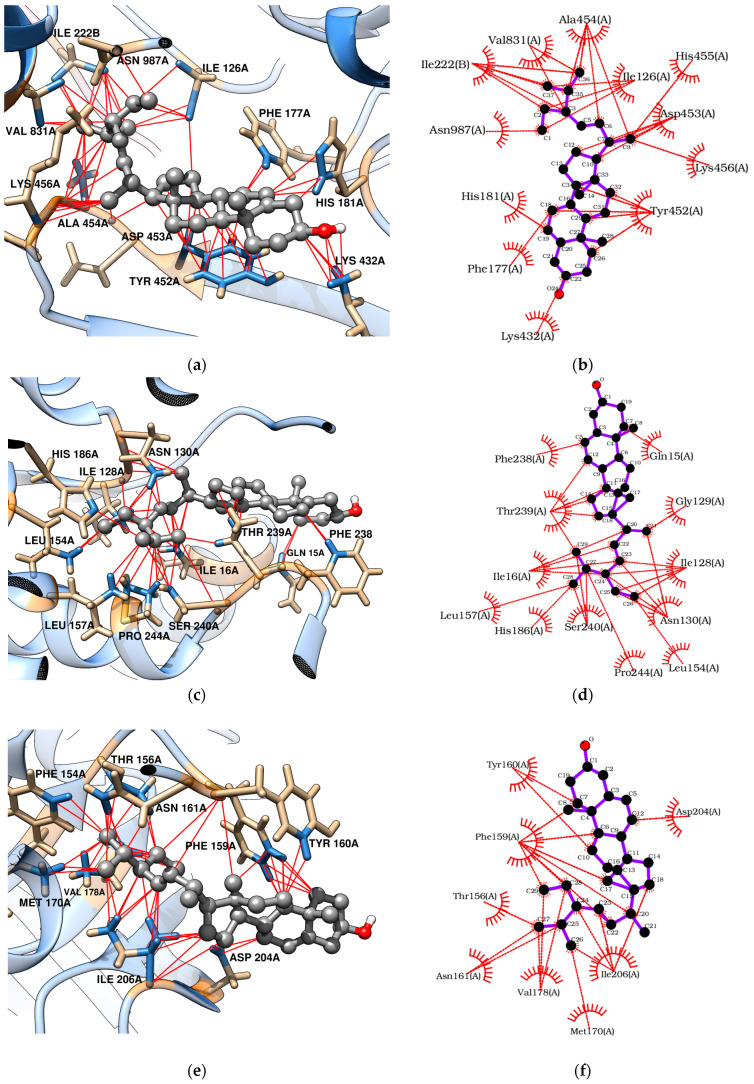
Molecular docking of stigmasterol with *T. vaginalis* proteins: (**a**,**b**) TvpFOR; (**c**,**d**) TvLDH; (**e**,**f**) TvPNP.

Docking analyses revealed that triterpenes, especially stigmasterol, interact favorably with TvLDH and TvpFOR, showing binding energies of −7.83 and −8.07 kcal/mol, respectively. These interactions are mainly hydrophobic in nature [54,55,56]. Betulin, however, may additionally form four conventional hydrogen bonds with key residues in the TvpFOR active domain (Figure 3b,d and Figure 4b,d).

In silico results also indicated that betulin and stigmasterol interact with Phe159, Val178, and Ile206 key residues located within the active site of the TvPNP enzyme (Figure 3f and Figure 4f) [52]. However, non-phytochemical ligands exhibited more exothermic docking scores, with values exceeding −7.19 kcal/mol. In contrast, TvMGL does not appear to represent a promising molecular target, as none of the triterpenes evaluated in this study displayed binding poses within its active site.

## 3. Discussion

To contribute to the understanding and documentation of Mexican ethnopharmacognosy and the development of new antiprotozoal agents, the present study evaluated the anti-*Trichomonas vaginalis* activity of *Tagetes nelsonii*. In this context, *Tagetes* species have been traditionally used to treat parasitic infections: decoctions of aerial parts of *T. lucida*, *T. filifolia* and, *T. minuta* are employed against gastrointestinal diseases caused by helminths and protozoa [57,58,59]. Moreover, *T. minuta* infusions are traditionally used as vaginal washes for infected discharge [26]. Research on therapeutic activities of *T. nelsonii* is limited [60], although antimicrobial, antifungal, cytotoxic and wound healing activities have been reported [61,62,63].

To our knowledge, this study represents the first report of antiprotozoal activity for *Tagetes nelsonii* crude extracts. The extracts exhibited weak activity against *T. vaginalis* (IC_50_ = 153.2–157.1 μg/mL). Previous studies have demonstrated antiprotozoal activity in other *Tagetes* species: the ethyl acetate fraction exhibited moderate to low antiplasmodial efficacy (IC_50_ = 20.0 and 70.0 μg/mL against chloroquine-sensitive and resistant strains of *Plasmodium falciparum*, respectively) [64]. Methanol extract of *T. minuta* showed promising activity against *Trypanosoma brucei* and *Trypanosoma cruzi* trypomastigotes (IC_50_ = 2.2 ± 1.5 and 9.2 ± 1.9 μg/mL, respectively) and moderate activity against *Plasmodium falciparum* (IC_50_ = 14.0 ± 2.8 μg/mL) and *Leishmania infantum* amastigotes (IC_50_ = 30.1 ± 4.6 μg/mL) [65]. Petroleum ether, hidrolated ethyl acetate and dichloromethane extracts of *T. mendocina* completely lysed *Leishmania amazonensis* and *L. brasiliensis* promastigotes at 100 µg/mL [66]. In the presented in this study, bioassay-guided fractionation od *T. nelsonii* stem bark led to the isolation of two triterpenes with moderate anti-trichomonacidal activity.

Triterpenes are naturally occurring 30-carbon compounds formed by isoprene units, present as carboxylic acids, alcohols, or aldehydes. The main natural triterpenoids are cyclic derivatives [67]. Polycyclic triterpenes have been reported to exhibit pharmacological properties such as anticancer, anti-inflammatory, antioxidant, antimicrobial biofilm, antiviral, anti-hypertensive, anti-atherosclerotic, anti-insulin resistance hepatoprotective, and immunomodulatory activity [68,69,70,71,72,73,74,75,76]. To date, at least 258 steroids and triterpenes from natural and synthetic sources have demonstrated antiprotozoal or anthelmintic activity [77]. In this study, betulin and stigmasterol exhibited moderate anti-*T. vaginalis* activity, with IC_50_ values of 54.5 and 43.8 μg/mL, respectively.

Betulin is a pentacyclic triterpene alcohol of the lupane series, characterized by a five- membered ring and an α-isopropenyl group at C-19 [44]. In contrast, stigmasterol is a tetracyclic unsaturated sterol (stigmasta 5,22-diene-3β-ol) with an ethyl group at C-24 of the side chain [78,79]. Both, stigmasterol and lupane-type triterpenes, have been found in *Tagetes* species [42,80,81]. Previous reports indicate antiprotozoal activity for botulin, with IC_50_ < 26.6 µg/mL againststandard and resistant strains of *Trypanosoma brucei* and *Trypanosoma congolense,* 35% inhibition at 22 µg/mL against *Leishmania donovani* axenic amastigotes, and IC_50_ = 58.8 µg/mL against *Leishmania infantum* promastigotes [82,83,84]. Stigmasterol has shown weak anti-trypanosomal activity (IC_50_ > 100 µg/mL) and promising activity (IC_50_ < 15 µg/mL) against *T. brucei* and *L. donovani* in a β-sitosterol mixture [46,49,85]. The lipophilicity of triterpenes may facilitate interactions with cell membranes and intracellular targets, inducing morphological changes on mitochondrial membrane, decreased membrane potential, which could explain the lack of hemolytic activity observed in this work [83].

To explore the putative mechanism of action of triterpenes, molecular docking was performed with enzymes required for *T. vaginalis* survival [10,50,51,52]. Both betulin and stigmasterol bound strongly to the TvpFOR substrate/cofactor pocket, predominantly via hydrophobic interactions. Stigmasterol interacts mainly with residues Ile126, Phe177, His181, Tyr452, Ala454, Val831, and Asn987 of chain A, whereas betulin additionally formed hydrophobic contacts with Thr34 and Arg117 and hydrogen bonds with Thr38, Glu41, Tyr127, and Asp453 (Figure 3b and Figure 4b). Structural characterization of a homologous TPP enzyme–pyruvate complex [55] shows electrostatic interactions of the pyruvate carboxylate with the guanidinium group of Arg114, hydrogen bonding of its oxygen atoms with Thr31, Asn996, and the TPP cofactor, and hydrophobic contacts between the substrate’s methyl group and the side chains of Ile123. The equivalent residues in TvpFOR (Thr34, Asn987 and Ile126) are involved in betulin interactions, suggesting that this triterpene interacts within the conserved catalytic pocket.

The hydrogenosome harbors an electron transport protein complex functionally linked to the TvPFOR-Ferredoxin system [10,86]. Previous studies have shown that compounds like metronidazole typically interact with Ferredoxin protein residues near the [2Fe–2S] cluster, such as Thr37, Leu31, and Lys46 to achieve proper alignment for electron transfer [86]. In contrast, stigmasterol in this study appears to adopt a different binding orientation, this alternative mode suggests that this triterpene may modulate enzyme activity through steric or allosteric effects rather than mimicking the electron-accepting behavior of nitro groups.

Regarding TvLDH, the aliphatic chain of stigmasterol appears to serve as the main contact region within the enzyme active site [87]. Hydrophobic interactions were observed between Ile16 and carbons C-20 to C-24, as well as between Ile128 and carbons C-23 to C-26. Additional contacts were identified involving Pro244 with C-27, the amino group of Asn130 with C-21, C-27 and C-28, and the imidazole group of His186 and the oxygen of hydroxyl group of Ser240 residue with C-25 to C-27, respectively (Figure 4c,d). Considering the central roles of PFOR and LDH in energy metabolism, these docking profiles support the hypothesis that disruption of these pathways contributes to the reduced *T. vaginalis* viability, especially by stigmasterol.

Key interactions were also observed between the triterpenes and the hydrophobic purine-binding site of TvPNP enzyme. Betulin engaged the A, B, and C rings, as well as the C-23/C-24 and C-30 positions, established hydrophobic interactions primarily with Phe159, while Ile206 interacted with the A ring and the C-23/C-24 carbons. In contrast, stigmasterol showed hydrophobic contacts between its B ring and C-19 with Phe159, whereas the aliphatic side chain interacted with Ile206 residues within the active pocket (Figure 4e,d). In TvPNP, the purine base is stabilized within the active site by four hydrophobic residues Phe159, Val178, Met180, and Ile206. The binding profiles coincide with those described in previous docking studies of TvPNP [87]. Among them, Phe159 and Val178 establish hydrophobic interactions with the purine ring, while the side chains of Ile206 and Met180 contribute additional van der Waals contacts near the 6-amino group of the base [52]. These findings suggest that triterpenes may modulate the purine salvage pathway, which is essential for maintaining purine nucleotide pools required for parasite survival.

Finally, the in silico interactions of the triterpenes with TvPNP and TvLDH were more favorable than those observed with metronidazole [87]. In addition, previous studies with natural oleanane-type inhibitors of *T. vaginalis* enzymes TvLDH and TvPNP showed binding scores higher than those observed in this study. However, aurones and lignans have demonstrated more promising docking affinities with TvPNP [88,89].

## 4. Materials and Methods

### 4.1. General Experimental Procedures

NMR spectra were obtained on Bruker AVANCE 500/600 MHz spectrometers (Bruker, Billerica, MA, USA) using standard pulse sequences, with CDCl_3_ as solvent and TMS as internal standard. TLC analyses were visualized under UV light or with cobalt chloride reagent after heating.

### 4.2. Plant Material

Mature stems of *Tagetes nelsonii* were collected in winter 2021 from Zinacantán, Chiapas, Mexico, identified botanically, and a voucher specimen (025883) was filed at the FCB Herbarium, Universidad Autónoma de Nuevo León. The plant material was cleaned, air-dried, and cut into small pieces.

### 4.3. Extraction and Isolation

Extracts were prepared for the evaluation of anti-*Trichomonas vaginalis* activity and hemolytic activities, 340 g of mature stems of *T. nelsonii* were sequentially macerated with *n*-hexane and methanol for three weeks at 25 °C. The solvent was replaced weekly (3 × 800 mL). Before filtration, each macerate was subjected to sonication in a low-power ultrasonic bath (Ney Ultrasonik (Cincinnati, OH, USA), 60 Hz, 15 min). The *n*-hexane and methanolic extracts were then concentrated under reduced pressure using a rotary evaporator.

Based on extraction yield and bioactivity, the methanolic extract (9.18 g) was partitioned with dichloromethane (CH_2_Cl_2_), yielding an active soluble fraction (271.6 mg) and an inactive insoluble fraction (8.90 g). In parallel, 1.30 g of the *n*-hexane extract was chromatographed on a Sephadex LH-20 column (Sigma, 30 × 7 cm) using *n*-hexane:CH_2_Cl_2_:methanol (2:1:1, *v*/*v*/*v*) eluent, affording four fractions. The most active fraction, with the highest yields (F2) was further separated on a normal-phase silica gel column (25 × 5 cm) with an ethyl acetate/*n*-hexane gradient, affording two pure compounds: compound 1 (15 mg) and compound 2 (7.8 mg). The remaining fractions were discarded either due to their low activity or low yield.

### 4.4. In Vitro Anti-Trichomonas Vaginalis Assays

*T. vaginalis* GT15 (CIBIN-IMSS) trophozoites were cultured axenically in TYI-S-33 medium with 10% bovine serum and used in the exponential growth phase. For in vitro antiprotozoal assays, 1 × 10^5^ trophozoites were incubated for 24 h at 37 °C with varying concentrations of extracts or fractions (17.5–300 μg/mL) dissolved in DMSO.

Pure compounds were tested at 50, 35, 25, 15, and 10 μg/mL, dissolved in absolute ethanol. Metronidazole (analytical grade, Sigma-Aldrich Corp., St. Louis, MO, USA) was included as positive control, while the blank consisted of culture medium containing trophozoites only. After incubation, trophozoites were detached by chilling, and a 1:10 dilution was prepared in formalin. Trophozoite density was measured using a hemocytometer, and IC_50_ values were calculated by probit analysis. Two independent experiments were performed in triplicate.

### 4.5. Hemolytic Activity of Extracts, Partitions and Compounds

Hemolytic activity was evaluated by monitoring the lysis of a 5% (*v*/*v*) erythrocyte suspension prepared in PBS solution (0.01 M, pH 7.4). Human erythrocytes were collected from healthy donors using EDTA, washed, and incubated with extracts, fractions, or pure compounds (100–500 μg/mL) for 30 min at 37 °C. Hemolysis was assessed spectrophotometrically at 540 nm, with distilled water as a positive control. All procedures were conducted under an approved ethical protocol by the Department of Chemistry, FCB/UANL (protocol No. ATL-06-2022, May 2022), with informed consent and strict confidentiality.

### 4.6. Target Proteins

In the absence of an experimentally determined *T. vaginalis* pyruvate:ferredoxin oxidoreductase (TvPFOR) crystal structure, a 3D model of the enzyme was generated using the AlphaFold3 server [90], based on the UniProt entry Q27088 (TvpFORA) [91]. The resulting model was subjected to structural optimization and validation using the MolProbity server [92]. The model showed a MolProbity score of 1.37 (98th percentile), a clashscore of 6.72 (88th percentile), with 98.35% of residues in favored regions and only 0.17% in disallowed regions of the Ramachandran plot. Additional validation metrics included 0.58% poor rotamers, no Cβ deviations, and 0.05% bad angles, indicating excellent stereochemical and geometric quality suitable for subsequent structural and functional analyses. The predicted protein model was further confirmed using the SWISS-MODEL v.2024-10.3 server [93]. For comparative docking analyses, the crystallographic structures of *T. vaginalis* lactate dehydrogenase (TvLDH, PDB ID: 5A1T), methionine gamma lyase (TvMGL, PDB ID: 1E5E), and purine nucleoside phosphorylase (TvPNP, PDB ID: 1Z36) were retrieved from the Protein Data Bank [94].

### 4.7. Molecular Docking

Molecular docking studies were conducted using the CB-DOCK2 server [95] and UCSF Chimera v.1.18 software [96]. The phytochemical ligands were retrieved from the ZINC20 database [97] (ZINC3978650, ZINC4096712) and the PubChem database [98] (CID 5280794, CID 72326). Two-dimensional ligand structures were converted into three-dimensional formats and geometrically optimized using Avogadro2 v.1.99.0 software [99]. Ligand preparation included the merging of non-polar hydrogen atoms and the assignment of Gasteiger partial charges using AutoDock Tools v.1.5.7p1 [100].

Target protein structures were cleaned by removing ligands, ions, and water before docking. The docking grid was defined based on the coordinates of the co-complexed ligands or active site residues. Docking conformations were ranked according to their binding energy scores and the best docking poses were chosen based on the lowest binding energy and an RMSD cutoff of 2.0 Å. Protein–ligand interactions were visualized and analyzed using LigPlot+ v.2.3.1 software [101].

To validate the molecular docking protocol, redocking assays were conducted using co-crystallized ligands from three Trichomonas vaginalis enzymes: oxamate (OXM) with lactate dehydrogenase (TvLDH, PDB: 5AT1), formycin (FMC) with purine nucleoside phosphorylase (TvPNP, PDB: 1Z36), and the PLP–PPG adduct (PPJ) with methionine γ-lyase (TvMGL, PDB: 1E5E). The predicted binding affinities were −5.09, −7.69, and −8.7 kcal/mol, with RMSD values of 1.20, 1.29–1.33, and 1.13 Å, respectively, compared to their crystallographic poses. For pyruvate:ferredoxin oxidoreductase (TvPFOR), whose structure was modeled via AlphaFold3, docking was validated using the thiamine pyrophosphate (TPP) cofactor from a homologous PFOR (PDB: 1B0P), yielding a binding affinity of −9 kcal/mol while preserving key catalytic interactions.

### 4.8. Statical Analysis

Anti-*T. vaginalis* activity was expressed as mean ± SD from two independent assays. To measure anti-*T.vaginalis* activity, the concentration of extract at which the parasite growth was inhibited by 50% (IC_50_) was calculated by Probit regression, and statistical differences among extracts, partitions, and compounds were analyzed using the paired Wilcoxon test with a significance level of *p* < 0.05.

## 5. Conclusions

This study reports, for the first time, the antiprotozoal potential of *Tagetes nelsonii* and its metabolites. Two triterpenes, betulin and stigmasterol, were identified with promising in vitro and in silico *activity* against *T. vaginalis*, with negligible hemolytic activity at 100 μg/mL. Altogether, these findings expand the structural framework of potential TvpFOR, TvLDH, and TvPNP inhibitors, and support the further optimization of triterpenoid compounds for the development of novel anti-trichomonal agents.

## Figures and Tables

**Table 1:** ^1^H- and ^13^C- NMR spectral data of terpenes isolated from *Tagetes nelsonii*.

Position	Betulin (1)				Stigmasterol (2)	
	Type	δ_C_ *	δ_H_ (*J* in Hz)	Type	δ_C_	δ_H_ (*J* in Hz)
1	CH_2_	32.7	2.04, m	CH_2_	37.4	1.01, m
2	CH_2_	25.1	1.68, m	CH_2_	31.5	1.85, m
3	CH	78.9	3.18, dd (4.7, 11.6)	CH	71.7	3.52, m
4	C	37.1	-	CH_2_	42.3	2.23, m
5	CH	55.2	1.41, m	C	140.7	-
6	CH_2_	18.2	1.51, m	CH	121.6	5.36, dd (2.3, 5.1)
7	CH_2_	34.2	1.39, m	CH_2_	31.8	1.85, m
8	C	40.8	-	CH	31.5	1.83, m
9	CH	50.4	1.25, m	CH	50.1	1.01, m
10	C	37.1	-	C	36.4	-
11	CH_2_	20.9	1.67, m	CH_2_	21.1	1.52, m
12	CH_2_	25.6	1.66, m	CH_2_	39.5	2.05–2.02, m
13	CH	38.0	2.03, d	C	42.2	-
14	C	42.8	-	CH	56.8	1.02, m
15	CH_2_	27.39	1.87, m	CH_2_	24.2	1.60, m
16	CH_2_	29.3	1.47, m	CH_2_	29.2	1.70, m
17	C	47.9	-	CH	55.9	1.13, m
18	CH	48.3	1.29, m	CH_3_	12.1	0.69, s
19	CH	50.4	2.37, td, (5.9, 11.1, 11.1)	CH_3_	19.6	1.01, s
20	C	150.9	-	CH	39.7	2.02, m
21	CH_2_	29.8	1.18, m	CH_3_	21.1	1.02, s
22	CH_2_	39.9	1.66, m	CH	138.2	5.15, dd (8.7, 15.2)
23	CH_3_	27.9	1.03, s	CH	129.2	5.02, dd (8.7, 15.2)
24	CH_3_	15.3	0.94, s	CH	51.2	1.51, m
25	CH_3_	16.0	0.83, s	CH	31.8	1.46, m
26	CH_3_	15.9	1.01, s	CH_3_	21.1	0.85, m
27	CH_3_	14.6	0.97, s	CH_3_	18.7	0.84, t
28	CH_2_	63.2	3.64, td (1.2, 6.7, 6.7)	CH_2_	24.9	1.69, m
29	CH_2_	109.2	4.69, d (2.5) 4.57, dd (1.5, 2.7)	CH_3_	11.8	0.80, m
30	CH_3_	19.3	1.65, s			

* NMR spectra (600 MHz, 150 MHz, CDCl_3_), δ in ppm.

**Table 2:** Anti- *T. vaginalis* and hemolytic activities of extracts and isolated compounds from *Tagetes nelsonii*.

Sample	Anti *T. vaginalis* Activity		Hemolytic Activity
	IC_50_ (µg/mL)	IC_50_ (µM)	IC_50_ (µg/mL)
Methanolic extract	157.1 ± 8.9		>500
DCM soluble part	131.0 ± 2.0		>500
DCM insoluble part	>200		>500
Hexanic extract	153.2 ± 9.0		>500
Betulin	54.5 ± 4.2	123.1 ± 9.5	>100
Stigmasterol	43.8 ± 4.3	100.6 ± 10.4	>100
Metronidazole	0.14 ± 0.04	0.8 ± 0.2	NT

DCM: dichloromethane; IC_50_ is expressed as the mean ± standard deviation; *n* = 3, NT = no tested. (*p* < 0.05).

**Table 3:** Predicted binding energies and molecular interactions between triterpenoids from *T. nelsonii* and *T. vaginalis* protein targets.

Compound	Target Protein	Binding Energy (kcal/mol) ^1^	Binding Score RMSD	Hydrogen Bond Residues (Length Å)	Non-Covalent Interactions
Betulin	TvpFOR	−8.04 ± 0.28	0.58 ± 0.82	Chain A: Thr38 (2.99), Glu41 (3.09), Tyr127 (3.15), Asp453 (3.20).	Chain A: Thr34, Asp99, Arg117, Ile126, Tyr127, Phe177, His181, Tyr 452, Ala454, Lys456, Val831, Phe857, Asn987, Thr988. Chain B: Ile222, Glu226.
	TvLDH	−6.61 ± 0.65	1.65 ± 0.14	None	Gln15, Ile16, Arg48, Ser88, Pro90, Leu91, Leu100, Asn130, Phe238, Thr239.
	TvPNP	−6.58 ± 0.66	1.58 ± 0.11	None	Thr156, Phe159, Tyr160, Asn161, Met170, Ala167, Val178, Asp204, His205, Ile206, His209.
Stigmasterol	TvpFOR	−8.07 ± 0.32	1.21 ± 1.71	None	Chain A: Ile126, Phe177, His181, Lys432, Tyr452, Asp453, Ala454, Lys456, Val831, Asn987. Chain B: Ile222.
	TvLDH	−7.83 ± 0.59	1.59 ± 0.29	None	Gln15, Ile16, Ile128, Asn130, Leu154, Leu157, His186, Phe238, Thr239, Ser240, Pro244.
	TvPNP	−7.19 ± 0.78	1.59 ± 0.23	None	Thr156, Phe159, Asn161, Ala167, Val178, Asp204, Ile206.

^1^ Media ± DS: TvpFOR, *n* = 2; TvLDH, *n* = 4; TvPNP, *n* = 5.

## Data Availability

The original contributions presented in this study are included in the article. Further inquiries can be directed to the corresponding author.
